# Unraveling the Secrets of Colistin Resistance with Label-Free Raman Spectroscopy

**DOI:** 10.3390/bios12090749

**Published:** 2022-09-11

**Authors:** Dimple Saikia, Priyanka Jadhav, Arti R. Hole, Chilakapati Murali Krishna, Surya P. Singh

**Affiliations:** 1 Department of Biosciences and Bioengineering, Indian Institute of Technology Dharwad, Dharwad 580011, India; 2 Tata Memorial Centre, Advanced Centre for Treatment Research and Education in Cancer, Navi Mumbai 410210, India; 3 Training School Complex, Homi Bhabha National Institute, Anushakti Nagar 400094, India

**Keywords:** Raman spectroscopy, antimicrobial resistance, colistin, microbiology, principal component analysis

## Abstract

The rise in number of infections from multidrug-resistant (MDR) Gram-negative microbes has led to an increase in the use of a variety of ‘polymyxins’ such as colistin. Even though colistin is known to cause minor nephro- and neuro-toxicity, it is still considered as last resort antibiotic for treating MDR infections. In this study, we have applied Raman spectroscopy to understand the differences among colistin sensitive and resistant bacterial strains at community level. We have successfully generated colistin resistant clones and verified the presence of resistance-causing MCR-1 plasmid. A unique spectral profile associated with specific drug concentration has been obtained. Successful delineation between resistant and sensitive cells has also been achieved via principal component analysis. Overall findings support the prospective utility of Raman spectroscopy in identifying anti-microbial resistance.

## 1. Introduction

The rapid and inappropriate use of antibiotics has led to a steep rise in the rate of antimicrobial resistance (AMR), which is now considered as the third most common cause of death and expected to have an enormous economic burden. It is predicted that, by the year 2050, worldwide, a total of 2.4 million people will succumb to death due to infections caused by drug resistant microbes. In the year 2017, the World Health Organization (WHO) provided a list of pathogens, stratified into critical and high priority groups based upon the AMR incidence rate and the urgency of the need to develop diagnostic and alternate therapeutic tools [1]. *Enterobacter* microbes resistant to last-resort antibiotics, such as colistin and carbapenems, are part of the critical group. These antibiotics are known to be extremely useful for treating multi-drug resistant (MDR) gram-negative infections [1,2].

Colistin has been used clinically since the 1960s for the treatment of infectious diseases caused by Gram-negative bacteria. However, due to toxicity, it was replaced by potentially less-toxic aminoglycosides and other antipseudomonal agents [3]. As cases of AMR have witnessed a sharp increase in recent time, the utility of this antibiotic has garnered significant attention, especially for tackling infections caused by MDR gram negative strains. It is known to bind to the lipopolysaccharides (LPS) and phospholipids in the outer membrane of the microbes [4,5]. This is followed by disruption of the membrane via replacement of divalent cations such as Ca^2+^ or Mg^2+^ from the membrane lipids, causing a leakage of cellular contents and ultimately death of the bacteria. The susceptibility breakpoints for colistin varies by bacterial species. For examples, microbes such as *Salmonella*, *Shigella* and *Escherichia coli* from the *Enterobacteriaceae* family are known to have a susceptibility and resistance breakpoints at <2 mg/L and >2mg/L, respectively [6,7,8]. Colistin resistance induction often occurs via LPS modification in the outer membrane. Novel moieties such as 4-amino-4-deoxy-L-arabinose (L-Ara4N), phospho-ethanolamine (PEtN), or galactosamine are added to the lipid A or the core of LPS. This reduces the overall negative charge from the phosphate residues and leads to a reduction in colistin affinity [4,9]. Some recent studies have identified MCR-1 plasmid mediated colistin resistance in *Escherichia Coli*. These genes are known to encode enzyme phospho-ethanolamine transferases which adds PEtN at the phosphate moiety in the lipid A molecules of the outer membrane [10,11,12]. As mentioned, this modification causes a reduction in the colistin affinity and imparts resistance. The resistance caused by mcr-1 gene has been shown to be widely disseminated in *E. coli* isolates from human, animals, food and the environment [13]. Global actions focused on accurate identification of colistin resistant microbes, preferably in a fast and objective manner, are extremely important in order to reduce the AMR burden [5]. A variety of detection methods ranging from traditional microbiology to molecular biology are reported in the literature. Broth microdilution, disk diffusion, Epsilometer test and Polymerase Chain Reaction (PCR) are some of well-known methods for identifying antibiotic resistant microbes [14,15]. Even though these methods have significantly improved the diagnostic potentials, they suffer from limitations in terms of high time consumption, cost and observer disagreements. Therefore, a rapid, sensitive and objective method to diagnose AMR is highly warranted. 

The above-mentioned limitations can be effectively neutralized by the application of optical technologies such as fluorescence, Raman, or infra-red spectroscopy in microbiology [16,17]. Fluorescence based methods have been applied for identification of microbes but overlapping signals originating from endogenous fluorophores such as flavins or aromatic amino acids have hindered its prospective utility for routine applications. Similarly, contamination due to strong water absorption in infra-red spectroscopy makes it unsuitable for a variety of in vitro or in vivo applications [18,19]. Raman spectra are generated via inelastic scattering of photons and are known to provide a biochemical fingerprint without any external reagents or sample preparation [20]. The spectral differences can be analyzed by appropriate ‘pattern recognition tools’ in an objective manner. Raman spectra are not influenced by water and have been utilized for variety of microbiology applications ranging from species level identification to in situ microbial growth, AMR identification etc. [21,22,23]. A recent study focused on rapid screening of resistant gram-negative bacteria using Raman spectroscopy coupled with hierarchical cluster analysis was reported by Lin et al. [24]. Nakar et al. also demonstrated that resistant *E. coli* strains have higher nucleic acid and protein content, and this leads to classification by trained machine learning model [25]. Colistin resistant *Klebsiella pneumoniae* was discriminated using SERS-based (Surface-enhanced Raman spectroscopy) biosensor coupled with machine learning techniques [26]. A recent study reported the feasibility of discrimination between sensitive and colistin resistant *E. coli* strains [27]. This study involves silver nanoparticle based SERS measurements to delineate spectral signals specific for colistin resistance and explored the feasibility of classification using machine learning. A primary drawback of these studies is inclusion of additional sample processing and treatment steps. Further, these studies do not provide any information on the major biochemical changes, due to colistin induction occurring at the community level in the natural environment. To overcome these limitations, in the present study we have performed community level spontaneous Raman measurements to identify major biochemical and biophysical changes associated with induction of colistin resistance in *E. coli* cells. We have also demonstrated the feasibility of the classification among strains resistant to different concentrations of colistin.

## 2. Materials and Methods 

### 2.1. Bacterial Strain

The colistin-sensitive bacteria *E. coli* (ATCC 11775) was obtained from the National Centre for Microbial Resource (NCMR), Pune, India. Phenotypic characterization, antimicrobial susceptibility testing (AST) and 16S ribosomal RNA sequencing (~1200–1500 bases) for species identification were performed. Bacteria were inoculated in LB-broth (Sigma-Aldrich, St. Louis, MI, USA) and stored in 50% glycerol at −80 °C until used. 

### 2.2. Induction of the Colistin Resistance

MCR-1 plasmid carrier in DH5alpha was obtained from Addgene, New Delhi, India. The heat-shock method was used for transforming the MCR-1 plasmid in calcium chloride treated *E. coli* cells. Transformed cells were screened on Kanamycin plates. After 18 h of incubation, positive colonies were utilized for plasmid isolation and confirmation. MCR-1 plasmid isolation was carried out using miniprep kits from QIAGEN India Pvt Ltd., New Delhi, India. Purity was established through spectrophotometer and gene size was confirmed by agarose gel electrophoresis.

### 2.3. Validation of Sensitive and Resistance Breakpoints

Minimal inhibitory concentration (MIC) for colistin sensitive *E. coli* cells was determined using the broth microdilution method as per The European Committee on Antimicrobial Susceptibility Testing (EUCAST) guidelines. The MIC is the lowest concentration with no visible growth. Colistin plates were prepared at the corresponding concentrations to verify the bacterial growth. MCR-1 transformed *E. coli* cells were initially incubated overnight at 37 °C. Microbes in the exponential phase of the growth (0.7–0.8 OD) were added to colistin (3.9 µg/mL) containing LB broth. This step was repeated 4–5 times until stable mutant colonies were obtained. The same protocol was performed for obtaining mutant cells resistant to 5.0, 6.5, 7.8 µg/mL colistin. It was found that all the mutant strains demonstrated slow growth as compared to the sensitive strain. Overall growth trend for resistant strains growing under mentioned colistin concentration was determined by performing optical density measurements at 4, 6, 12, 18, 24, 36, 48, 60, and 78 h time points. All the mutant strains were stored in 50% glycerol at −80 °C until used. 

### 2.4. Rapid Polymyxin NP (Nordmann/Poirel) Test

The colistin-sensitive strains were inoculated in LB-broth, while the mutant strains were inoculated in LB-broth with colistin at different concentrations (3.9, 5.0, 6.5, 7.8 µg/mL) and were incubated overnight at 37 °C. Rapid NP solution was prepared with LB broth, phenol red (Sigma Aldrich, St. Louis, MI, USA) and D-glucose at pH ~6.5. Colistin-NP solution was prepared by adding antibiotics with rapid NP solution at previously mentioned antibiotic concentrations. One hundred and fifty (150) µL of colistin-free rapid NP solution was added in A1 followed by adding 150 µL of colistin-NP solutions in the B1. Bacterial cell suspension with matching optical density between 3–3.5 was prepared by diluting, using saline. Fifty (50) µL of saline solution was added in the A1 and B1 wells with no cells. Fifty (50) µL of sensitive cells were added in A2. Similarly, 50 µL resistant cells were added in A3–A6 and B3–B6, in the increasing order of colistin concentration. The plate was incubated at 37 °C without cover for 2 h. 

### 2.5. Raman Spectroscopy 

As mentioned, colistin-sensitive and resistant *E. coli* strains have different generation times. To avoid any spectral variation due to differences in the growth stage, both resistant and sensitive cells were collected at the logarithmic phase. This translated in to collection of colistin sensitive cells at 12 h and resistant strains at 12, 18, 24, 36 h depending upon the antibiotic concentration. Cell number was adjusted by saline dilution to match the optical density. The culture medium was centrifuged at 12,000 RPM for 10 min at 4 °C and the supernatant was discarded. The cells were washed three times with saline. The pellet was transferred to the CaF_2_ window for acquiring spectra using Raman alpha 300R (WITec, GmbH) microscope, equipped with a laser of 532 nm wavelength, 100× objective (Zeiss, NA = 0.8), 300 mm spectrograph with 600 g/mm grating and charged-coupled detector (CCD). Spectra were acquired with an integration time of 5 s with 10 accumulations. Spectra were collected at different spots from each pellet and repeated thrice to ensure reproducibility. 

### 2.6. Spectral Pre-Processing and Analysis

The pre-processing steps included baseline correction, filtering and normalization of the Raman spectra. Spectra were interpolated between 500–1800 cm^−1^. The cosmic background was removed by filtering. The pre-processed spectra obtained from colistin-resistant and sensitive cells were used as input to explore the possibility of classification using Principal Component Analysis (PCA). The input data consisted of control (14 spectra), 3.9 µg/mL (30 spectra), 5.0 µg/mL (28 spectra), 6.5 µg/mL (33 spectra) and 7.8 µg/mL (24 spectra). All the corrections and analyses were performed using in-house MATLAB programs. Scatter plots were generated using open source Orange data mining software.

### 2.7. Field Emission Scanning Electron Microscopy (FESEM) 

The morphology of the sensitive and resistant strains was examined using Carl Zeiss Gemini 300 FESEM. Briefly, the sample preparation protocol included collection of 500 µL cell suspension from the log phase. Cell pellet was collected after centrifugation. Cells were fixed using 4% paraformaldehyde (PFA) and washed with phosphate buffer saline. Five (5) µL of cells were placed on the aluminium stub using double-side carbon tape and then allowed to dry followed by coating with a 20 nm thick layer of gold. Morphologies were analyzed under standard conditions. 

## 3. Results 

### 3.1. Generation and Validation of Colistin Resistant E. coli Cells

**Figure 1:**
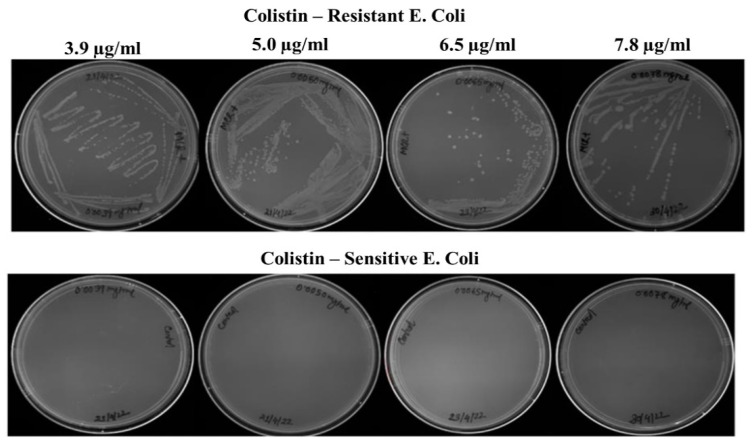
MCR-1 transformed strains with different concentration of colistin (3.9, 5.0, 6.5, and 7.8 µg/mL).

Broth microdilution assays revealed that colistin concentration above 1.9 µg/mL leads to inhibition of growth in sensitive microbes. Therefore, this concentration was considered as the sensitivity breakpoint for *E. coli* cells used in this study. Bacteria growing any concentration above 1.9 µg/mL can be considered as resistant. After MCR-1 transformation, the bacterial growth was assessed in concentrations above 1.9 µg/mL colistin. As shown in Figure 1, clear growth of MCR-1 transformed bacterial cells was observed at 3.9, 5.0, 6.5, 7.8 µg/mL colistin concentrations. Colistin antibiotic is known to be a poor diffuser in the agar media [28,29]. This limits the applicability of ‘disk diffusion and E- test’ for validating presence of colistin resistant microbes.

**Figure 2:**
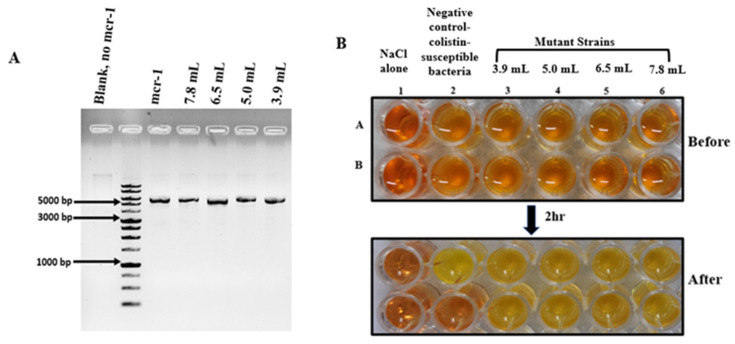
(**A**) Agarose gel image showing the presence of the MCR-1 plasmid in resistant strains (~5081 bp) (**B**) Rapid polymyxin NP test based identification of colistin sensitive and resistant strains.

Presence of the MCR-1 in the transformed bacteria was further confirmed by plasmid extraction, Figure 2A. The rapid polymyxin NP test is known to be extremely sensitive in rapidly differentiating sensitive and resistant enterobacterial microbes. The test is based on glucose metabolization activity of resistant microbes in presence of colistin. The formation of acid in resistant species is identified by a visible color change from orange to yellow of pH indicator phenol red [30,31]. As shown in Figure 2B, wells A1 and B1 with NaCl remains orange after 2-h incubation, suggesting no contamination. Wells from A2–A6 and B2–B6 contain bacterial suspension. The color change from orange to yellow was observed in A2, containing sensitive strain growing in absence of colistin. In contrast B2 has no change of color after incubation as no metabolic activity of the sensitive microbes is expected in presence of colistin. Wells from A3–A6, B3–B6 contain resistant strain and therefore color change is observed in presence or absence of colistin. These results were validated by two independent observers unaware of the type of the isolate. 

### 3.2. Resistant Induction

**Figure 3:**
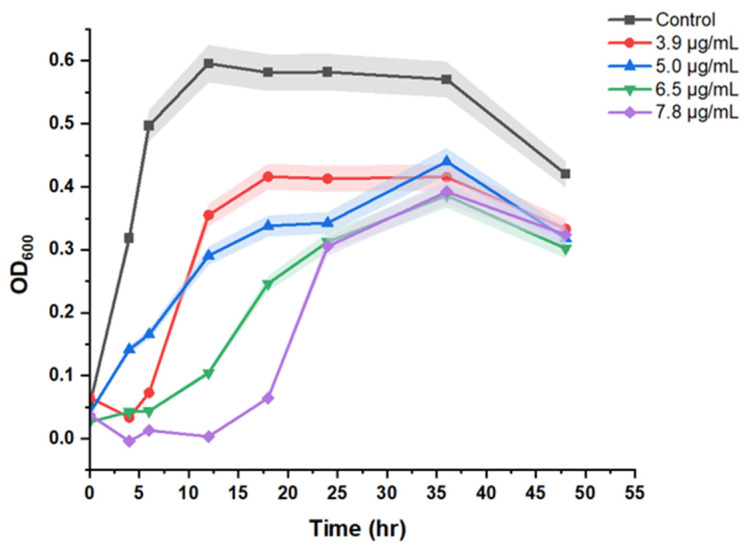
Growth pattern comparing sensitive and resistant strains.

After confirming the MCR-1 plasmid incorporation, growth kinetics study of sensitive and resistant microbes over a period of 48 h was performed. As shown in Figure 3, in contrast to control, the overall growth trend of colistin resistant microbes is different. The primary difference was noted in the time to reach the ‘exponential’ or ‘log’ phase. In control or sensitive cells, the ‘log phase’ started at 6 h post inoculation. However, it started at 12 h for cells resistant to 3.9 and 5 µg/mL concentration of colistin. Similarly, the ‘log’ phase initiation time for cells resistant to 6.5 µg/mL colistin was 18 h, while this was 24 h for cells resistant to 7.8 µg/mL concentration of colistin. This is an important aspect to be considered before starting the spectroscopic measurements, as metabolic activity of cells can introduce spectral differences. In one of our recent studies, we demonstrated that, based upon the Raman metabolic fingerprint, bacterial cells at different phases of growth cycle can be successfully discriminated [32]. To rule out any differences due to variations in the metabolic stage, the bacterial cells in the present study were collected at the mentioned time points corresponding to ‘logarithmic phase’. The number of cells was also kept constant across all measurements by keeping the same optical density for every spectral acquisition.

### 3.3. Spectral and Statistical Data Analysis

**Figure 4:**
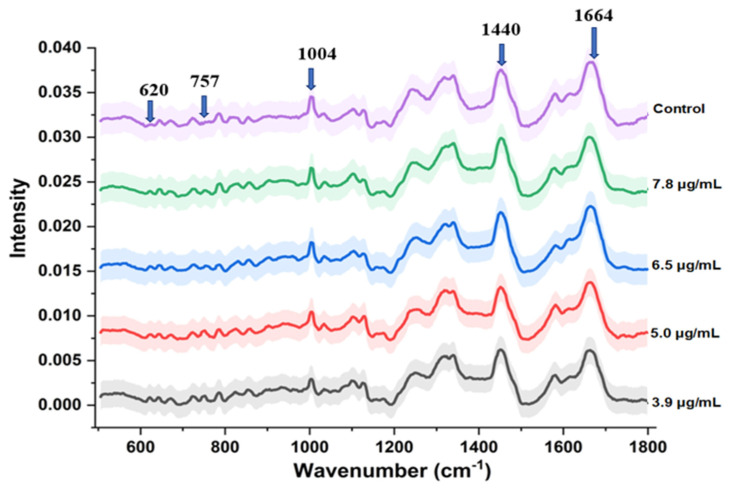
Average Raman spectra from colistin sensitive and resistant bacterial cells collected from the log phase. The shaded area represents the standard deviation. Characteristic spectral bands are marked with an arrow.

Average Raman spectra from colistin sensitive and resistant bacterial cells is shown in Figure 4. Spectral bands corresponding to phenylalanine (1003 cm^−1^) and nucleic acids originating from phosphate back bone (784 cm^−1^) and guanine residue (1580 cm^−1^) can be clearly seen. In addition to these, bands at 1130, 1244, and 1333 cm^−1^ originating from phospholipid residues were also observed. Origin of the strongest band at 1440 cm^−1^ has been attributed to CH_2_ stretching/CH_3_ asymmetric deformation of lipids. Intensity of bands assigned to nucleic acids (784 cm^−1^) and amide I (1664 cm^−1^) was found to be similar in sensitive and resistant strains. A closer view of the spectral profile reveals a unique band in resistant strains at 757 cm^−1^. Origin of this band has been attributed to Ethanolamine functional group [33]. As mentioned, the colistin resistance in *E. coli* cells is induced primarily by modifications in the outer membrane that obstructs colistin to establish electrostatic contacts. Phospho-ethanolamine (PEtN) is one of the common moieties added to the lipid A molecules of the outer membrane [4,9]. The intensity of spectral bands in the range of 880–980 cm^−1^ was found to be higher in resistant cells. Primary Raman bands in this region belong to glycogen, amino acids such as proline, hydroxyproline and valine. As the cationic antimicrobial peptide resistance (colistin) is known to be assisted by thickening of capsular layers via deposition of additional polysaccharide, increase in glycogen content can be expected [34]. Some additional bands at positions 620 cm^−1^ (C-C twist aromatic ring), 828 cm^−1^ (ring breathing tyrosine), 860, 1103 and 1740 cm^−1^ (polysaccharides) were also found to have higher intensity in resistance cells with respect to sensitive. Spectral assignments were performed as per the literature [35,36].

**Figure 5:**
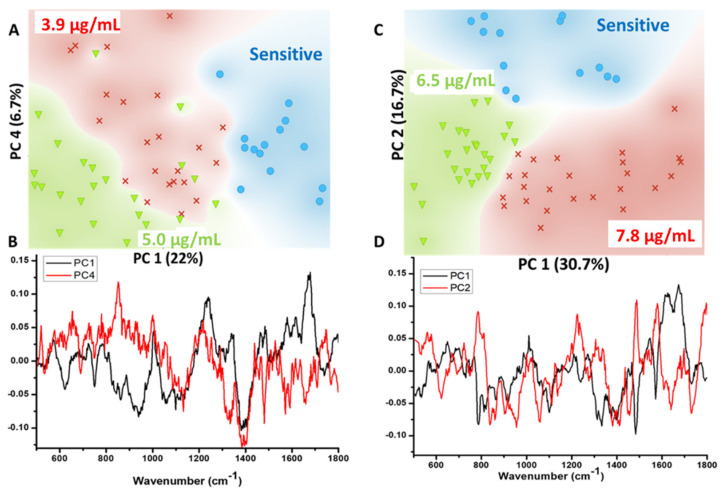
(**A**) PCA-scatter plot between sensitive and cells resistant to colistin concentration of 3.9 and 5 µg/mL (**B**) Loading plot of PC1- and PC4 used for classification. (**C**) PCA-scatter plot between sensitive and cells resistant to colistin concentration of 6.5 and 7.8 µg/mL (**D**) Loading plot of PC1- and PC2 used for classification.

In the next step, the feasibility of classification between sensitive and resistance strains was explored using Principal Component Analysis (PCA). To facilitate the visual discrimination between the groups, PCA was performed separately by comparing spectra from sensitive cells against strains resistant to colistin concentrations below or higher than 5 µg/mL. Scatter plots generated using principal components are shown in Figure 5.

**Figure 6:**
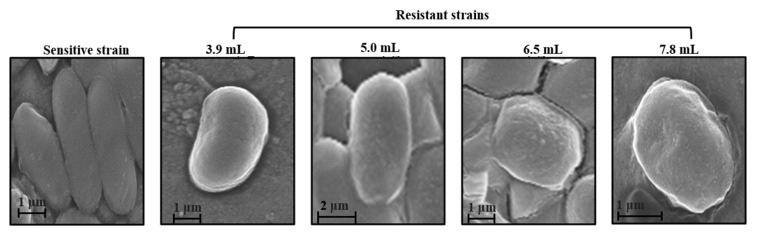
Morphological changes associated with induction of colistin resistance (3.9, 5.0, 6.5, and 7.8 ug/mL) analyzed using FESM.

Scatter plot specific for strains resistant to colistin concentration below 5 µg/mL demonstrate a minimal overlap among the resistant strains. However, the cluster of sensitive cells is exclusive. Loading plots of factor 1 (22%) and factor 4 (6.3%) are presented in Figure 5B. The loading profile of these factors further supports the already mentioned spectral differences in the mean spectrum. Clearly visible bands at 757 cm^−1^ attributed to the ethanolamine functional group and 620 cm^−1^ from aromatic amino acids can be clearly seen. In addition to these, bands at 860, 1103 and 1740 cm^−1^, all originating from polysaccharides, also seem to be a primary contributing factor in overall discrimination of the spectral data. In contrast to this, PCA of strains resistant to colistin concentration above 5 µg/mL revealed three exclusive clusters, Figure 5C. Loading profile of factor 1 (30.7%) and factor 2 (16.7%) used for generating the scatter plot is presented in Figure 5D. Spectral bands at 620, 757, 860, 1103 and 1740 cm^−1^ were observed. Overall, the spectral features and PCA are suggestive of successful delineation between colistin resistant and sensitive cells. Morphological changes associated with induction of colistin resistance were also analyzed using FESM. As shown in Figure 6, colistin resistant *E. coli* cells demonstrate a circular shape in contrast to rod-shaped sensitive strains [37]. 

## 4. Discussion

Antibiotics are considered as one of most valued accomplishments of modern medicine. They have helped humanity immensely to tackle life-threating infections. However, this advancement has been followed by acquisition of endurance or resistance in microbes to the antimicrobial activity of antibiotics [3]. Appearance of AMR is a serious concern for infections caused by Gram-negative microbes due to limited availability of antibiotics. Colistin is a multicomponent polypeptide antibiotic which is recommended for the treatment of multi drug resistant Gram-negative infections [28,38]. The existing methods for identification of resistant strains include assays such as phenotypic assays, staining, PCR, or immunoassays. These methods have primary limitations in terms of high time consumption and objectivity. Identification of colistin-resistant and *E. coli* strains in a rapid and objective manner can be helpful for designing appropriate therapeutic strategies. The rapid polymyxin NP test is one of the recently developed methods for identifying colistin resistance in microbes isolated from animals or humans [30]. As shown in Figure 2, resistant strains are metabolically active in presence of colistin. This leads to the formation of acids in the culture media which causes a color change of the red phenol indicator. The primary benefit of this approach is the short time (2 h) required for the read-out. Even though, this approach is sensitive towards identifying presence or absence of colistin resistant microbes, it has limited applicability in differentiating between microbes resistant to a specific concentration of colistin. This is an important aspect that needs consideration, especially for defining dose specific therapeutic interventions. 

Vibrational spectroscopic approaches are known to be sensitive to both morphological and biochemical variations associated with resistance induction and therefore can be prospectively utilized. One of the primary changes observed in colistin resistant cells is their higher tendency to form clumps. This is expected as the reduced charge is known to enhance the colloid aggregate stability due to diminished electrostatic repulsions [34,39]. In addition to the surface modification due to phospho-ethanolamine substitution, the morphological changes could also be a primary factor contributing to the overall spectral variations observed between sensitive and resistant cells, Figure 4. The origin of these changes can be assigned to the differences in glycan strand length distributions and cross-linking density. Lack of pili or extracellular appendages in the colistin resistant population could also be another factor contributing to the observed spectral differences [34,40]. 

PCA is one of the most widely utilized unsupervised multivariate analysis methods for overviewing of the spectral data. It also helps in the identification of outliers. The method works by transforming the variables (wavenumbers) into uncorrelated variables, also called as principal components (PCs), for dimensionality reduction without influencing the overall variance in the spectral data. Loading plots or profile of these components can be used for identifying the primarily contributing spectral bands. The PC-1 explains maximum variation followed by successive PCs covering the remaining variability [41]. The scatter plots shown in Figure 5 clearly demonstrate the feasibility of discrimination between not only the resistant and sensitive cells overall but also among concentration specific resistant cells. 

Soon et al. using atomic force microscopy demonstrated multiple changes in the cell wall of Gram-negative *Acinetobacter baumannii* associated with colistin resistant induction [40]. Their findings suggest that, in contrast to rod-like shapes of sensitive strains, colistin resistant microbes demonstrate a circular pattern. Our preliminary investigations using FESM corroborated these findings. As shown in Figure 6, *E. coli* cells with induction of colistin resistance start to acquire a spherical shape. A closer analysis of the surface topography of the resistant cells revealed higher surface roughness in resistant cells with respect to sensitive strains. This can further lead to reduced membrane fluidity causing an increase in colistin resistance ability. 

New omics technologies have provided a tool to map or identify different kind of microbially controlled processes. However, techniques which can provide this information in a non-destructive manner are still highly warranted. Raman micro-spectroscopy methods are unique in the sense that they offer a non-destructive tool for investigating microbial metabolism in situ. The uniqueness of our study lies in the fact that spectral measurements were performed at the community level, which is closer to the natural environment of the microbes. With further technical and data analysis advancement, we can assume that Raman spectroscopy based methods will have a widespread adoption soon, especially for microbiological applications. 

## 5. Conclusions

The findings of the study support another facet of Raman based approaches for prospective applications in anti-microbial resistance monitoring. Scanning electron micrographs revealed biophysical changes due to induction of colistin resistance. Prospectively it can offer an alternate or adjunct tool for enabling better, rapid and label-free monitoring of colistin related AMR diagnostics. Further studies with larger bacterial cohorts and greater diversity are required to generate a concentration and strain specific database that can be used for identifying resistant strains under clinical settings. This can help in better management that can in turn help improve diagnostics to assist the fight against the rise of AMR and, ultimately, improve global health. 

## Data Availability

Not applicable.
